# Nearest-Neighbor Interactions and Their Influence on the Structural Aspects of Dipeptides

**DOI:** 10.1155/2013/939865

**Published:** 2013-09-18

**Authors:** Gunajyoti Das, Shilpi Mandal

**Affiliations:** Department of Chemistry, North Eastern Hill University, Shillong 793022, India

## Abstract

In this theoretical study, the role of the side chain moiety of C-terminal residue in influencing the structural and molecular properties of dipeptides is analyzed by considering a series of seven dipeptides. The C-terminal positions of the dipeptides are varied with seven different amino acid residues, namely. Val, Leu, Asp, Ser, Gln, His, and Pyl while their N-terminal positions are kept constant with Sec residues. Full geometry optimization and vibrational frequency calculations are carried out at B3LYP/6-311++G(d,p) level in gas and aqueous phase. The stereo-electronic effects of the side chain moieties of C-terminal residues are found to influence the values of Φ and *Ω* dihedrals, planarity of the peptide planes, and geometry around the C_7_ *α*-carbon atoms of the dipeptides. The gas phase intramolecular H-bond combinations of the dipeptides are similar to those in aqueous phase. The theoretical vibrational spectra of the dipeptides reflect the nature of intramolecular H-bonds existing in the dipeptide structures. Solvation effects of aqueous environment are evident on the geometrical parameters related to the amide planes, dipole moments, HOMOLUMO energy gaps as well as thermodynamic stability of the dipeptides.

## 1. Introduction

**Figure 1:**
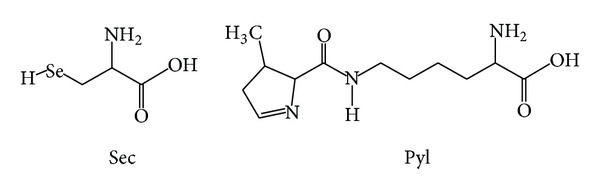
Chemical structures of selenocysteine (Sec) and pyrrolysine (Pyl).

Generally, twenty canonical amino acid residues adequately build up the proteins and enzymes necessary to support most of the cellular functions in all the three domains of life on earth. Selenocysteine (Sec) and pyrrolysine (Pyl) are the two rarely occurring genetically encoded amino acids whose presence in the active sites of some enzymes enables them to sustain life in some extraordinarily unique ways [1–7]. The chemical structures of Sec and Pyl are portrayed in Figure 1. Although the distribution of Pyl is limited to methanogenic archea and certain bacteria, Sec is commonly found in eubacteria, archaea, and eukarya [5]. Sec and Pyl are cotranslationally inserted into proteins corresponding to UGA (opal codon) and UAG codons (canonical stop codon) respectively, which are generally responsible for terminating the process of protein biosynthesis.

 The dynamic properties and functional specificity of the proteins and polypeptides are known to depend primarily on the linear sequence of amino acid residues [8]. Therefore, over the last few decades, small amino acid sequences like di- or tripeptides have been used extensively as model systems in the experimental and theoretical studies concerning the structure of proteins and energetics of protein folding. On the other hand, since theoretical or computational approaches are difficult to employ directly to the large macromolecular systems such as polypeptides or proteins, model systems serve as an easy and computationally less expensive alternate way to understand the structure of protein. Some of the most important structural features of the protein backbone have been reproduced theoretically by considering dipeptides as model systems. It is now realized that computational techniques are indispensable in elucidating atomic level structural information about biologically active molecules [9–11]. 

 The gas-phase structural studies on dipeptides provide us the opportunity to understand their intrinsic properties free from the solvent or crystal phase effects. In gas phase, the structural features of the dipeptides mainly depend on a delicate balance between the stabilizing intramolecular H-bonds, destabilizing repulsive lone electron pairs, and steric effects. Gas phase structural studies on dipeptides arising from the genetically encoded amino acids [12–15] have pointed out that in most of the dipeptides the amide plane are not completely planar; and this has been explained in terms of the cumulative effect of steric hindrance of –R group and H-bonding. However, it is of fundamental importance to determine the conformational details of a biologically important molecule in aqueous solution since most of the biochemical processes occur in an aqueous environment. 

 The solvent effects play crucial role in shaping the secondary structure of proteins by modifying the interplay between intra- and intermolecular H-bond interactions existing in the primary amino acid sequences. The effects of solvation on the conformations and energies of dipeptides have been well documented in literature [16–23]. In these studies the energetics and structural features of the dipeptides in gas and solvent phases are analyzed to understand the effect of the surrounding environment on the stabilities and conformational preferences of the dipeptides. In a strong polar solvent like water, the interactions among the nearest-neighbor residues of the dipeptides are dramatically modified as compared to those in gas phase, which consequently affect the Ramachandran dihedrals (*ψ*, *ϕ*) [24, 25] and confer markedly different conformations to the dipeptides in the aqueous phase. Investigations of the numerous parameters involved in dipeptide structure prediction have now been regarded as a pivotal part of the computational studies concerning the structure of protein and energetics of protein folding [26].

**Figure 2:**
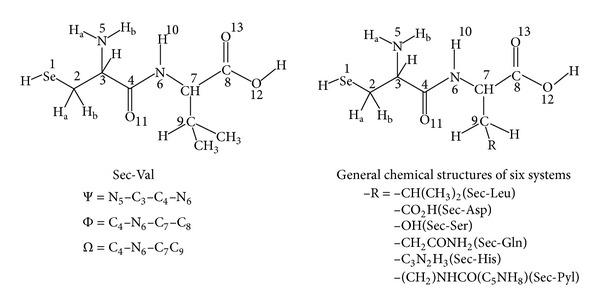
Schematic representation of chemical structures of the seven dipeptides studied.

 In this study, efforts are being made to examine the effects of solvation and identity of the varying C-terminal residue on the energetics, structural features of the peptide planes, geometry about the *α*-carbon atoms, values of the *ψ* and *ϕ* dihedrals, theoretically predicted vibrational spectra, dipole moments, rotational constants, HOMO/LUMO energies as well as their energy gaps, and types of intramolecular H-bonding interactions that may play crucial roles in determining the structure and stability of a series of dipeptides whose C-terminal positions are varied with seven different amino acid residues namely valine (Val), leucine (Leu), aspartic acid (Asp), serine (Ser), glutamine (Gln), histidine (His), and pyrrolysine (Pyl), while the N-terminal position is kept constant with a selenocysteine (Sec) residue. All these amino acid residues are taken as neutral (nonionic) species. The standard three letter abbreviations are used to represent an amino acid while a particular dipeptide is named by listing the N-terminal residue first. Thus, Sec-Val dipeptide corresponds to a structure in which Sec is in the N-terminal position and Val is in the C-terminal position.  Figure 2 schematically represents the chemical structures of all the seven dipeptides. The C_4_–N_6_ is the peptide bond of a given dipeptide while C_3_ and C_7_ are the *α*-carbon atoms of its N- and C-terminal residues respectively. To facilitate a clear representation of the intramolecular hydrogen bond interactions present in the dipeptides some of the hydrogen atoms are named as H_a_ or H_b_. This theoretical structural study on the seven dipeptides in gas as well as in simulated aqueous phase is expected to provide the opportunity to know the influence of local interactions on the structural aspects of dipeptides at an atomic level which in turn may help us to understand the dynamics and functional specificity of proteins and polypeptides and in enhancing this rapidly expanding area of research.

## 2. Computational Methodology

The molecular geometries of all the selected dipeptides were subjected to full geometry optimization and vibrational frequency calculations using the B3LYP/6-311++G(d,p) level of theory [27, 28] of Gaussian 03 package [29]. The efficacy of B3LYP/6-311++G(d,p) in studying conformational behavior and various other properties of amino acids has been explained in the literature [30]. The computations were conducted in gas as well as in aqueous phase using a polarizable continuum model (PCM) [31]. The accuracy of self-consistent reaction field (SCRF) model in predicting the structure and energetics of dipeptides has already been justified in literature [32]. Absence of imaginary frequency value in the vibrational frequency calculations proved the optimized geometries to be true minima. Zero point energy (ZPE) corrections were applied to the total energies of all the dipeptides using a correction factor 0.9877 [33]. The theoretically predicted vibrational frequencies were scaled with appropriate scaling factors (1.01 for below 1800 cm^−1^ while 0.9679 for above 1800 cm^−1^) [33]. Use of diffuse functions is important to take into account the relative diffuseness of lone pair of electrons when a molecule under investigation contains lone pair of electrons [34] while polarization functions are useful in studying the conformational aspects where stereoelectronic effects play an important role [35].

## 3. Results and Discussion

**Figure 3:**
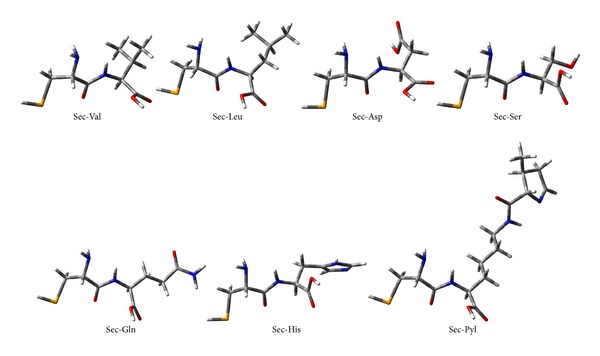
Optimized structures of the dipeptides in aqueous phase.

**Figure 4:**
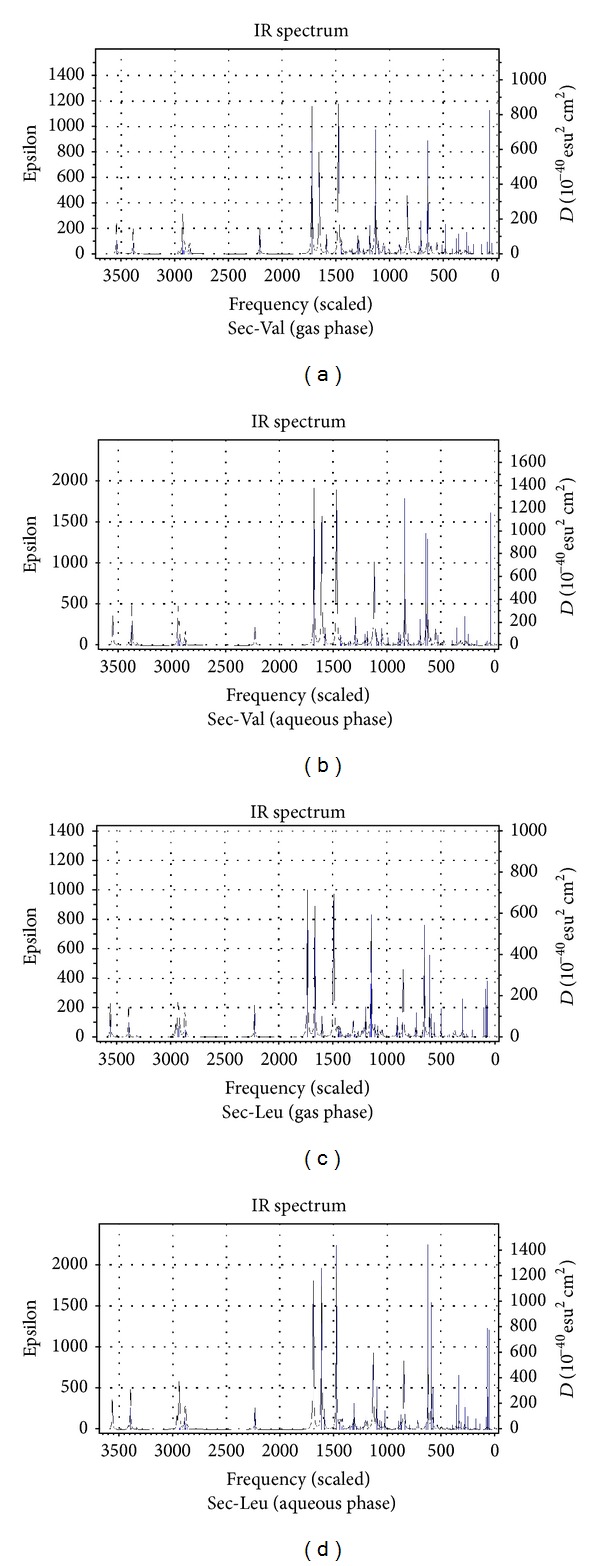
Vibrational spectra of Sec-Val and Sec-Leu in gas and aqueous phase.

**Figure 5:**
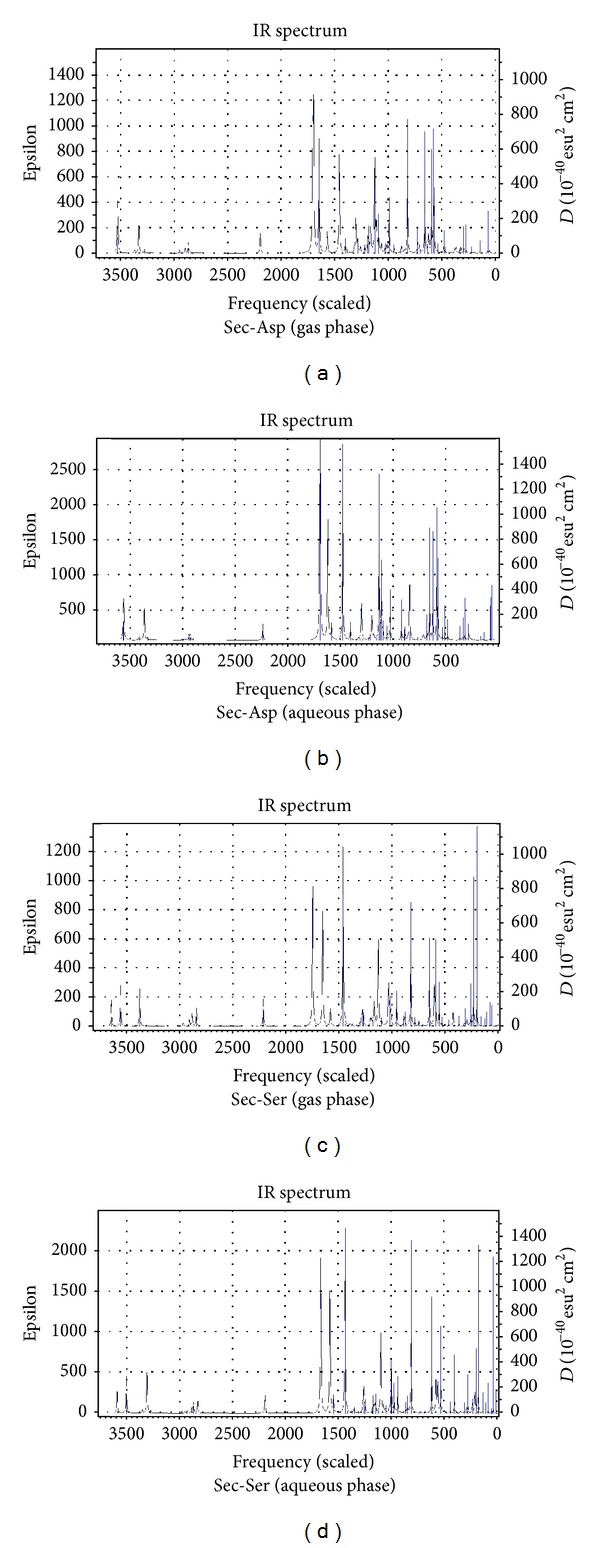
Vibrational spectra of Sec-Asp and Sec-Ser in gas and aqueous phase.

**Figure 6:**
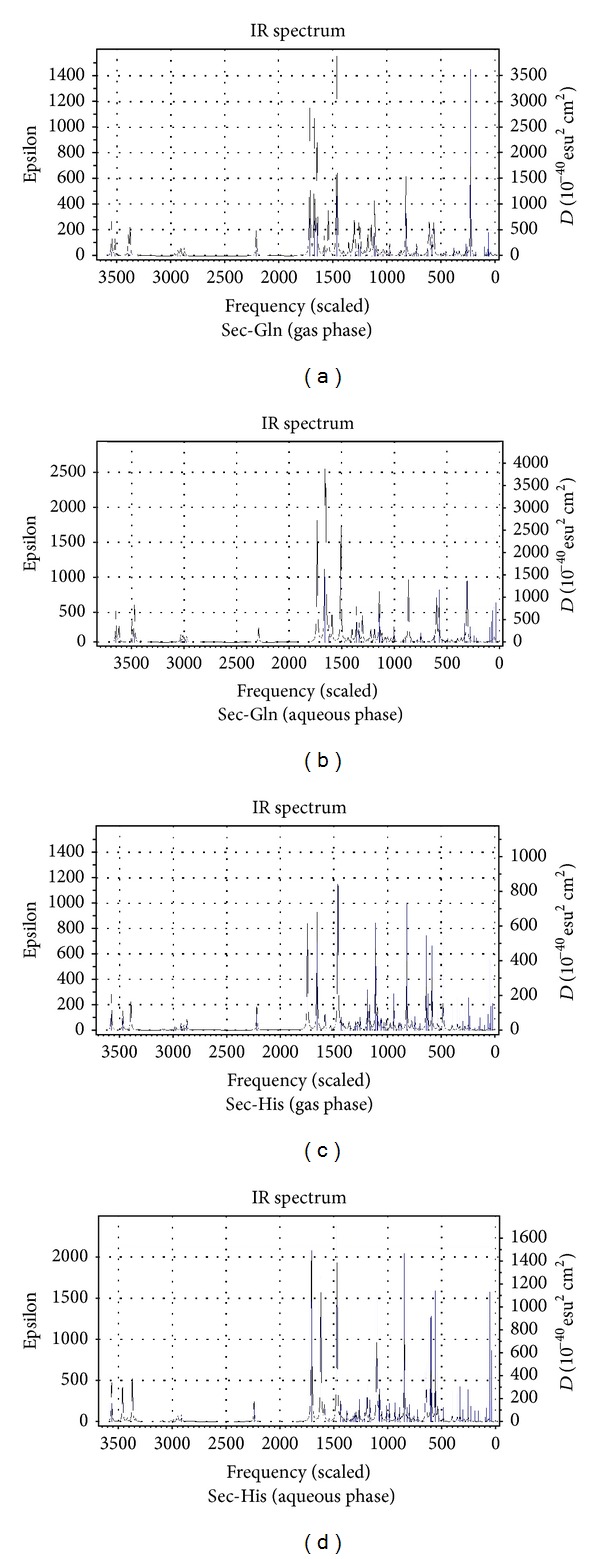
Vibrational spectra of Sec-Gln and Sec-His in gas and aqueous phase.

**Figure 7:**
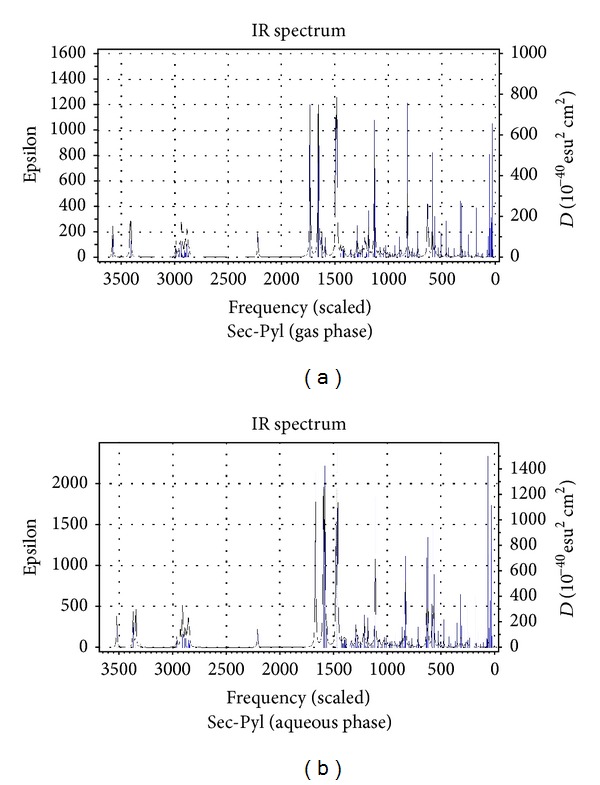
Vibrational spectra of Sec-Pyl in gas and aqueous phase.

Table 1 presents the gas and aqueous phase data on total energies, rotational constants, and dipole moments of the dipeptides calculated at B3LYP/6-311++G(d,p) level of theory. Tables 2 and 3 list the values of the bond lengths and bond angles of the amide planes of the dipeptides respectively (the gas phase values are given in brackets).  Table 4 collects the values of the four dihedral angles considered to monitor the planarity of the peptide planes of the dipeptides (viz. C_3_–C_4_–N_6_–C_7_, O_11_–C_4_–N_6_–H_10_, C_3_–C_4_–N_6_–H_10_ and O_11_–C_4_–N_6_–C_7_), the values of *Ω* (C_4_–N_6_–C_7_–C_9_) dihedrals considered to specify the orientations of the side-chain moieties, and the values of the two well-known Ramachandran backbone dihedral angles *ψ* (N_5_–C_3_–C_4_–N_6_) and *ϕ* (C_4_–N_6_–C_7_–C_8_) which are useful in studying the effects of solvation on the dipeptide structures as well as in predicting the overall structure of proteins.  Table 5 represents the gas and aqueous phase data on the geometrical parameters considered to examine the geometry around the *α*-carbon atoms.  Table 6 lists some important intramolecular H-bonding interactions that play crucial roles in the energetics and in conferring the observed conformations to the dipeptides in both the phases. Some of the characteristic frequency and intensity values (given in brackets) of the dipeptides calculated at the B3LYP/6-311++G(d,p) level of theory are given in Table 7.  Table 8 represents the DFT results on the HOMO/LUMO energies, and their energy gaps for the dipeptides in both phases.  Figure 3 represents the optimized structures of the dipeptides in aqueous phase while Figures 4, 5, 6, and 7 represent the theoretical IR spectra of the seven dipeptides both in gas and aqueous phase (scaled with a correction factor 0.9679).

### 3.1. Structure and Stability of the Dipeptides

All the seven dipeptide geometries exhibit large values of total dipole moments (listed in Table 1), ranging from 2.910 to 9.218 D in gas phase and from 5.423 to 12.542 D in aqueous phase, which indicate that they have greater polar character and consequently possess greater affinity to polar solvents. Thus, the data on the total energy of dipeptides correctly predicts that the dipeptide geometries are thermodynamically more stable in a strong polar solvent such as water than in gas phase by an energy difference that may range from 12.07 to 19.60 kcal/mol. The accuracy of DFT method in predicting the rotational constants of conformers of some aliphatic amino acids has been discussed in the literature [36, 37]. In the absence of any experimental data on rotational constants and dipole moments, these theoretically predicted values may assist experimentalists in determining the other conformers of the seven dipeptides studied here. 

 The gas and aqueous phase bond length values of the five bonds of the amide planes that is, C_3_–C_4_, C_4_=O_11_, C_4_–N_6_, N_6_–H_10_ and N_6_–C_7_, listed in Table 2, suggest that very little variance in the bond length values of the amide plane results as the identity of the C-terminal residue of a given dipeptide changes. Maximum deviations of 0.006 Å in gas phase and 0.004 Å in aqueous phase from their respective average values indicate that the bond lengths are essentially fixed. However, due to solvation effects the aqueous phase bond length values of the above mentioned bonds deviate from their respective gas phase values. For example, in aqueous phase, the exposed C_4_=O_11_ and N_6_–H_10_ bonds are elongated up to 0.009 and 0.002 Å respectively; whereas the buried C_4_–N_6_ bonds are shortened by a range of 0.007 to 0.011 Å for all the systems.  Table 3 lists the values of the six bond angles of the amide planes that is, C_3_–C_4_–O_11_, C_3_–C_4_–N_6_, O_11_–C_4_–N_6_, C_4_–N_6_–C_7_, C_4_–N_6_–H_10_ and H_10_–N_6_–C_7_; and the data in both the phases indicates very little changes in the bond angle values as the individuality of the C-terminal residue of the dipeptides changes. Maximum deviations of 1.3° in gas phase and 0.7° in aqueous phase indicate that the bond angles are also essentially fixed. The solvent effects on these bond angles are quite apparent when their aqueous phase data is compared with the corresponding gas phase values; a maximum deviation up to 2.0° is observed for C_4_–N_6_–C_7_ angle in the Sec-Asp.

 The predicted gas and aqueous phase values of the four dihedral angles of the dipeptides, namely, C_3_–C_4_–N_6_–C_7_, O_11_–C_4_–N_6_–H_10_, C_3_–C_4_–N_6_–H_10_, and O_11_–C_4_–N_6_–C_7_, listed in Table 4, provide valuable information regarding the planarity of the peptide planes. The values of the two dihedral angles C_3_–C_4_–N_6_–C_7_ and O_11_–C_4_–N_6_–H_10_ should be close to 180° and those for the other two, that is, C_3_–C_4_–N_6_–H_10_ and O_11_–C_4_–N_6_–C_7_ should be close to 0° if indeed the amide plane is planar. The data presented in Table 4 shows that in aqueous phase the values of the four dihedral angles deviate up to a maximum value of 4.7° from the expected value whereas in gas phase the maximum deviation observed is 11.4°. Thus, these dihedral angles do not deviate dramatically from their expected values in both phases, however, the extent of deviations observed in the values of the four dihedral angles obviously suggests that the geometry of the amide planes are not perfectly planar regardless of whether the systems are in gas phase or in strong polar solvents like water. It is expected that the conformations of the seven dipeptides predicted at B3LYP/6-311++G(d,p) level are reliable since it has been pointed out that full geometry optimization of gaseous tryptophan conformers at B3LYP/6-311G(d) and MP2/6-311++G(d,p) levels do not produce any noticeable structural changes, only the conformer energies change by small amounts [38]. Therefore, it is reasonable to assume that solvation effects cannot drastically improve the planarity of the amide planes, and the extent of the deviations from planarity primarily depends on two factors—(a) steric interactions of the side chain moieties of the C-terminal residues (–SC group) and (b) intramolecular H-bond formation by the H- and O-atoms of the amide planes with their adjacent moieties belonging to the C- and N-terminal residues. The intramolecular H-bond interactions that play crucial roles in deviating the amide planes from planarity and in imparting the observed conformations to the dipeptides in gas and aqueous phase are listed in Table 6, and a discussion on these interactions is also offered in a succeeding section of this paper.

Table 4 also lists the –SC groups of the C-terminal residues of the dipeptides as well as the gas and aqueous phase values of the *ψ*, *ϕ* and *Ω* dihedrals. Previous gas phase studies [12, 13] have pointed out that the value of *ϕ* increases as the size of a given –SC group increases. However, a thorough analysis of the dipeptide structures studied here reveals that both size as well as the type of functional groups present in a –SC group may influence the *ϕ* value and planarity of the amide plane of a given dipeptide. A large sized –SC group may compete for its physical space requirements to accommodate itself in between the amide plane and carboxylic group of the C-terminal residue of a given dipeptide and consequently influence the planarity of the amide plane. On the other hand, the –SC groups, depending on the type of functional groups present in them, may exert electrostatic repulsive or electrostatic attractive forces on their neighboring atoms belonging to the peptide planes and the carboxylic group of the C-terminal residues of the dipeptides which may also influence the values of *ϕ* as well as planarity of the amide planes. The gas and aqueous phase values of *ϕ* and *Ω* dihedrals of the dipeptides reveal that in solvent phase the type of functional groups present in the –SC groups is more important in influencing the *ϕ* and *Ω* values of the dipeptides rather than the size of the –SC groups. The polar solvents are known to leave remarkable influence on the conformational properties of dipeptides by weakening the intraresidue hydrogen bonds and leading to the appearance of new energy minima [19–21, 23]. Thus, the differences in the gas and aqueous phase values of *ϕ* dihedrals in Sec-Asp and Sec-Pyl systems, 32.8° and 35.5° respectively, can be justified on the basis of the type of functional groups present in the –SC groups of Asp and Pyl residues. Similarly, the smaller *ϕ* values of Sec-Gln than those of Sec-Ala (even though the –SC group of Gln is bigger in size than that of Val) and larger *ϕ* values of Sec-Ser than those of the other six systems (in spite of having smallest sized –SC group in its C-terminal residue) can be explained by invoking the influence of functional groups present in their respective –SC groups.

### 3.2. Geometry about the *α*-Carbon Atoms

Since the protein structures usually contain thousands of amino acid residues, the geometries about the *α*-carbon atoms of the individual residues play important role in deciding the overall structure of the proteins. The three bond angles considered to monitor the geometry around the C_3_ *α*-carbon atoms of the dipeptides are N_5_–C_3_–C_2_, N_5_–C_3_–C_4_ and C_2_–C_3_–C_4_, while N_6_–C_7_–C_8_, N_6_–C_7_–C_9_ and C_9_–C_7_–C_8_ are the same for the C_7_ atoms. The *α*-carbon atoms of the amino acids are sp^3^ hybridized and therefore the ideal bond angle should be 109.5°; however, this is not expected due to their stereogenic character. By monitoring the above mentioned bond angles around each *α*-carbon atom of the dipeptides, one can get an idea about how the change in identity of the C-terminal residue can affect the geometries about these *α*-carbon atoms. This DFT study also provides us the opportunity to probe the effects of solvation on the geometries of the *α*-carbon atoms.  Table 5 lists the gas and aqueous phase data on the bond angles about the *α*-carbon atoms. Maximum deviations of 0.2° in aqueous and 1.2° in gas phase from their respective average values suggest that the geometries about the C_3_ atoms do not change much with the change in the identity of the C-terminal residues. On the other hand, with maximum deviations up to 3.5° in aqueous and 3.6° in gas phase from their respective average values, the bond angles around the C_7_ change appreciably with the change in identity of the C-terminal residue of the dipeptides. These observations can be justified by invoking the two factors—size and the type of functional groups present in the –SC groups as previously mentioned while discussing the planarity of the peptide planes. The stereoelectronic effects of the varying –SC groups on the geometry of the C_3_ atoms are very little as they reside at a distance of four bonds away from these *α*-carbon atoms. On the contrary, since the varying –SC groups are situated adjacent to the C_7_ atoms, the geometry around them is affected by the changing identity of the –SC groups. The solvation effects are also more prominent on the geometry of the C_7_ atoms (a maximum deviation up to 2.4° is observed for the angle C_9_–C_7_–C_8_ in Sec-Pyl system) than that on the C_3_ atoms where the maximum deviation predicted is 1.8° for the N_5_–C_3_–C_4_ angle of Sec-Asp system.

### 3.3. Intramolecular Hydrogen Bond Interactions

The different conformers of a dipeptide molecule are known to be stabilized by a delicate interplay of different types of intramolecular hydrogen bonds (H-bonds) [15]. The strength of these H-bonds depends on two factors, (a) shorter is the distance A–H*⋯*B than the sum of their van der Waals radii and (b) closer the angle A–H*⋯*B to 180°, where A–H is H-bond donor and B is H-bond acceptor [22, 23].  Table 6 lists two types of intramolecular H-bonds, namely, N*⋯*H–N and O*⋯*H–C, whose interplay is very crucial in imparting the observed deviations of the peptide planes from planarity as well as in determining the energetics of the seven dipeptides. The gas phase intramolecular H-bond combinations of the dipeptides are similar to those in the aqueous phase. In aqueous phase, the B*⋯*H distances of N_5_ *⋯*H_10_–N_6_ bonds are shortened by a range of 0.019 to 0.077 Å while the same of the O_11_ *⋯*H–C_7_ bonds are elongated up to a magnitude of 0.204 Å. On the other hand, the gas and solvent phase data on the two H-bonds O_12_ *⋯*H–C_7_ and O_13_ *⋯*H–C_7_ clearly indicates the effects of size and the type of functional groups present in the –SC groups on the conformation of the dipeptides as well as on the number and type of H-bond interactions existing in the dipeptide molecules. For example, the absence of O_13_ *⋯*H–C_7_ and presence of O_12_ *⋯*H–C_7_ bonds, only in the case of Sec-Asp system, can be explained on the basis of the identity of the –SC group of Asp residue.

### 3.4. Calculated Vibrational Spectra

Table 7 lists the characteristic frequency and intensity (given in brackets) values of only those vibrational modes which are sensitive to the structural changes caused by the varying C-terminal residues and solvent effects. The theoretically predicted vibrational spectra of the seven dipeptides in both phases provide valuable information to understand the existence and nature of various types of intramolecular H-bonds in the dipeptides. It is evident from Table 7 that the vibrational frequencies shift invariably towards the lower side of frequency scale corresponding to the presence of intramolecular H-bond interactions. The shortening of N_5_ *⋯*H_10_–N_6_ bonds in aqueous phase structures is well reflected by the lowering in the frequency values of the *ν*(N_6_–H_10_) stretching by a range of 17 to 36 cm^−1^ than those in the gas phase. Solvent effects also lower the frequency values of the *ν*(C_4_=O_11_) modes of the dipeptides by a magnitude up to 52 cm^−1^ in aqueous phase which may be due to the elongation of bond lengths in solvent phase (the C_4_=O_11_ bonds are elongated up to 0.009 Å in the aqueous phase). The variations observed in *ν*(C_7_–H) stretching values can be attributed to the effects of the changing identity of the –SC groups of the C-terminal residues. The *ν*(C_3_–H) stretching values of the dipeptides remain relatively unchanged since the geometry around the C_3_ *α*-carbon atoms does not change much with the changes in identity of the –SC groups.

### 3.5. HOMO/LUMO Energies

Table 8 represents the DFT data on the highest occupied molecular orbital (HOMO), lowest unoccupied molecular orbital (LUMO) energies, and their energy gaps for the dipeptides in both the phases. These data suggest that the HOMO-LUMO energy gaps for the dipeptides increase in the presence of a solvent with high-dielectric constant than those in gas phase. This point has been well discussed in the literature [39]. In aqueous phase the predicted HOMO-LUMO energy gaps for dipeptides are (range from 5.4719 to 5.7745 eV) always higher than those in the gas phase (range from 5.1617 to 5.2532 eV) which indicate that the dipeptides are more stable in aqueous phase. Information obtained from HOMO-LUMO energy gaps has been used in elucidating the chemical activity [40–42] as well as in various other interesting physicochemical properties [43] of biologically important molecular systems. 

## 4. Conclusions

All the seven dipeptide geometries exhibit large values of total dipole moments, ranging from 2.910 to 9.218 D in gas phase and 5.423 to 12.542 D in aqueous phase, and as a consequence the aqueous phase structures show more thermodynamic stabilities than the gas phase structures by a range of 12.07 to 19.60 kcal/mol. The stereo-electronic effects of the varying –SC groups influence the values of *ϕ*, planarity of the peptide planes, and geometry around the C_7_ *α*-carbon atoms of the dipeptides while the solvation effects are evident on the values of bond lengths and bond angles of the amide planes. The geometry of the amide planes is not perfectly planar regardless of whether the systems are in gas or in strong polar solvents like water and the deviations from planarity primarily depends on two factors—(a) steric interactions of the side chain moieties of the C-terminal residues and (b) intramolecular H-bond formation by the H- and O-atoms of the amide planes with their adjacent atoms belonging to the C- and N-terminal residues. The gas phase intramolecular H-bond combinations of the dipeptides are similar to those in the aqueous phase; two types of intramolecular H-bonds (viz. N*⋯*H–N and O*⋯*H–C) play crucial roles in influencing the geometry of the peptide planes and in determining the energetics of the dipeptides. The variations in the values of *ν*(C_7_–H) stretching frequencies of the dipeptides reflect the effects of the changing –SC groups on the geometry around the C_7_ atoms, while the *ν*(C_3_–H) stretching values remain relatively unchanged since the geometry around the C_3_ *α*-carbon atoms are not affected by the changing identity of the –SC groups. The HOMO-LUMO energy gaps for the dipeptides are more in aqueous phase than those in gas phase indicating that the dipeptides are more stable in aqueous phase. 

## Supplementary Material

Cartesian coordinates of optimized structures of the dipeptides at b3lyp/6-311++g(d,p) in gas and aqueous phase.

## Figures and Tables

**Table 1:** Gas and aqueous phase data on total energies^a^ (kcal/mol), rotational constants (GHZ), and dipole moments (Debye) of the dipeptides calculated using B3LYP/6-311++G(d,p) level of theory.

Dipeptides	Phases	Total energies	Rotational constants			Dipole moments
			*A *	*B *	*C *	
Sec-Val	Aqueous	−1914673.58	0.89777	0.21696	0.20165	8.217
	Gas	−1914661.51	0.88925	0.21786	0.20379	5.408
Sec-Leu	Aqueous	−1939331.27	0.69533	0.19564	0.17033	8.749
	Gas	−1939318.66	0.66357	0.19800	0.17511	5.443
Sec-Asp	Aqueous	−1983716.72	0.63845	0.21115	0.18677	5.615
	Gas	−1983701.12	0.65542	0.22214	0.19679	2.910
Sec-Ser	Aqueous	−1912566.68	1.14681	0.21622	0.20891	10.530
	Gas	−1912551.23	1.13530	0.21713	0.20946	6.968
Sec-Gln	Aqueous	−1995899.84	0.90448	0.13379	0.12285	5.423
	Gas	−1995881.71	0.82585	0.13320	0.12498	3.165
Sec-His	Aqueous	−2006567.87	0.80904	0.12584	0.12281	12.542
	Gas	−2006548.27	0.70813	0.12993	0.12457	9.218
Sec-Pyl	Aqueous	−2201743.59	0.31784	0.04734	0.04349	8.428
	Gas	−2201725.10	0.24766	0.05474	0.04856	5.134

^a^ZPVE corrected; scaled with a correction factor 0.9877.

**Table 2:** Calculated bond lengths (in angstrom) for the peptide planes of the dipeptides; the gas phase values are given in brackets.

Dipeptides	C_3_–C_4_	C_4_=O_11_	C_4_–N_6_	N_6_–H_10_	N_6_–C_7_
Sec-Val	1.540 (1.541)	1.232 (1.223)	1.349 (1.358)	1.013 (1.011)	1.448 (1.448)
Sec-Leu	1.538 (1.541)	1.233 (1.224)	1.347 (1.356)	1.013 (1.012)	1.450 (1.448)
Sec-Asp	1.539 (1.540)	1.230 (1.221)	1.353 (1.364)	1.015 (1.014)	1.446 (1.446)
Sec-Ser	1.540 (1.543)	1.230 (1.221)	1.353 (1.361)	1.015 (1.013)	1.451 (1.455)
Sec-Gln	1.538 (1.540)	1.232 (1.223)	1.348 (1.357)	1.013 (1.012)	1.452 (1.448)
Sec-His	1.541 (1.543)	1.230 (1.221)	1.354 (1.361)	1.015 (1.013)	1.454 (1.456)
Sec-Pyl	1.539 (1.542)	1.233 (1.224)	1.348 (1.357)	1.014 (1.013)	1.449 (1.449)

Average	1.539 (1.541)	1.231 (1.222)	1.350 (1.359)	1.014 (1.013)	1.450 (1.450)
MD^a^	0.002 (0.002)	0.002 (0.002)	0.004 (0.005)	0.001 (0.002)	0.004 (0.006)

^a^Maximum deviation from average values.

**Table 3:** Calculated bond angles (in degrees) for the peptide planes of the dipeptides; the gas phase values are given in brackets.

Dipeptides	C_3_–C_4_–O_11_	C_3_–C_4_–N_6_	O_11_–C_4_–N_6_	C_4_–N_6_–C_7_	C_4_–N_6_–H_10_	H_10_–N_6_–C_7_
Sec-Val	121.2 (121.4)	114.8 (114.6)	123.9 (124.0)	123.5 (122.6)	115.3 (115.9)	121.1 (121.0)
Sec-Leu	121.4 (121.4)	115.4 (114.8)	123.2 (123.8)	122.6 (122.9)	116.3 (116.1)	121.0 (120.6)
Sec-Asp	121.6 (122.4)	114.6 (113.9)	123.8 (123.6)	123.4 (121.4)	115.6 (114.9)	120.9 (119.9)
Sec-Ser	121.5 (121.7)	114.4 (114.3)	124.1 (124.0)	123.6 (122.7)	115.7 (116.8)	120.7 (120.4)
Sec-Gln	121.8 (121.6)	115.2 (114.9)	122.9 (123.5)	122.5 (122.2)	116.5 (116.6)	121.0 (121.0)
Sec-His	121.6 (121.8)	114.3 (114.2)	124.1 (124.0)	123.8 (122.8)	115.6 (116.6)	120.5 (120.4)
Sec-Pyl	121.3 (121.4)	115.1 (114.5)	123.5 (124.1)	123.2 (123.0)	115.9 (116.3)	120.9 (120.6)

Average	121.5 (121.7)	114.8 (114.5)	123.6 (123.9)	123.2 (122.5)	115.8 (116.2)	120.9 (120.6)
MD^a^	0.3 (0.7)	0.6 (0.6)	0.7 (0.4)	0.7 (1.1)	0.7 (1.3)	0.4 (0.7)

^a^Maximum deviation from average values.

**Table 4:** Calculated dihedral angles (in degrees) of the dipeptides at B3LYP/6-311++G(d,p) level of theory; the gas phase values are given in brackets.

Dipeptides	–SC Groups	C_3_–C_4_–N_6_–C_7_	O_11_–C_4_–N_6_–H_10_	C_3_–C_4_–N_6_–H_10_	O_11_–C_4_–N_6_–C_7_	*ψ*	*ϕ*	Ω
Sec-Val	–CH(CH_3_)_2_	176.4 (171.9)	−178.8 (−178.9)	−0.5 (−0.3)	−1.9 (−6.6)	19.8 (28.4)	−95.8 (−103.7)	137.7 (129.3)
Sec-Leu	–CH_2_CH(CH_3_)_2_	176.4 (172.5)	−178.0 (−179.0)	0.4 (0.1)	−2.0 (−6.7)	18.3 (24.8)	−73.4 (−91.9)	162.9 (142.5)
Sec-Asp	–CH_2_CO_2_H	175.8 (−169.6)	−178.9 (169.0)	−0.6 (−11.4)	−2.6 (10.7)	20.6 (33.1)	−99.0 (−131.8)	135.7 (103.6)
Sec-Ser	–CH_2_OH	177.4 (176.5)	178.3 (174.1)	−3.5 (−7.7)	−0.8 (−1.7)	21.3 (30.3)	−118.4 (−135.7)	117.8 (100.6)
Sec-Gln	–(CH_2_)_2_CONH_2_	178.5 (175.3)	179.8 (−177.8)	−2.2 (1.2)	0.5 (−3.7)	22.2 (22.9)	−65.4 (−80.9)	170.8 (154.5)
Sec-His	–(CH_2_)_2_C_3_N_2_H_3_	178.4 (177.1)	177.1 (173.5)	−4.7 (−8.3)	0.2 (−1.2)	23.7 (30.7)	−108.5 (−131.4)	126.1 (103.9)
Sec-Pyl	–(CH_2_)_4_NHCO(C_5_NH_8_)	177.1 (174.7)	−178.1 (179.3)	0.5 (−2.2)	−1.4 (−3.8)	18.3 (27.2)	−83.0 (−118.5)	152.8 (117.4)

MD^a^		4.2 (10.4)	2.9 (11.0)	4.7 (11.4)	2.6 (10.7)			

^a^Maximum deviation from expected values.

**Table 5:** Calculated bond angles (in degrees) for the *α*-carbon atoms of the dipeptides; the gas phase values are given in brackets.

Dipeptides	*α*-carbon atoms C_3_			*α*-carbon atoms C_7_		
	N_5_–C_3_–C_2_	N_5_–C_3_–C_4_	C_2_–C_3_–C_4_	N_6_–C_7_–C_8_	N_6_–C_7_–C_9_	C_9_–C_7_–C_8_
Sec-Val	113.4 (114.5)	110.9 (110.4)	111.5 (110.9)	113.7 (113.5)	112.9 (113.5)	110.3 (110.2)
Sec-Leu	113.3 (114.2)	110.8 (110.6)	111.5 (111.0)	112.9 (113.3)	112.1 (112.8)	109.2 (109.8)
Sec-Asp	113.6 (114.6)	110.8 (109.0)	111.5 (110.6)	114.1 (111.9)	111.6 (112.6)	109.9 (110.1)
Sec-Ser	113.4 (114.5)	110.7 (110.2)	111.5 (110.8)	110.6 (109.5)	110.7 (110.9)	111.3 (111.7)
Sec-Gln	113.6 (114.3)	110.6 (110.6)	111.5 (110.9)	112.6 (113.5)	109.2 (109.9)	111.0 (110.7)
Sec-His	113.6 (114.5)	110.6 (110.2)	111.5 (110.9)	108.8 (108.3)	110.6 (111.2)	113.5 (113.0)
Sec-Pyl	113.3 (114.4)	110.9 (110.5)	111.5 (110.8)	113.7 (113.1)	111.3 (112.8)	109.5 (111.9)

Average	113.5 (114.4)	110.8 (110.2)	111.5 (110.8)	112.3 (111.9)	111.2 (112.0)	110.7 (111.1)
MD^a^	0.2 (0.2)	0.2 (1.2)	0.0 (0.2)	3.5 (3.6)	2.0 (2.1)	2.8 (1.9)

^a^Maximum deviation from average values.

**Table 6:** H-bond distances^a^ (in angstrom) for the intramolecular H-bond interactions detected in the dipeptide structures in both the phases.

Dipeptides	Phases	N_5_ *⋯*H_10_–N_6_	O_11_ *⋯*H–C_7_	O_12_ *⋯*H–C_7_	O_13_ *⋯*H–C_7_
Sec-Val	Aqueous	2.152	2.422	abs	2.658
	Gas	2.228	2.349	abs	2.712
Sec-Leu	Aqueous	2.175	2.595	abs	2.555
	Gas	2.208	2.413	abs	2.682
Sec-Asp	Aqueous	2.156	2.401	2.723	abs
	Gas	2.176	2.323	2.670	abs
Sec-Ser	Aqueous	2.147	2.443	abs	2.537
	Gas	2.224	2.402	abs	2.553
Sec-Gln	Aqueous	2.188	2.708	abs	2.532
	Gas	2.207	2.504	abs	2.599
Sec-His	Aqueous	2.157	2.438	abs	2.530
	Gas	2.221	2.412	abs	2.548
Sec-Pyl	Aqueous	2.162	2.523	abs	2.589
	Gas	2.215	2.366	abs	2.637

^a^Only the (B*⋯*H) distances are listed where B is H-bond acceptor; abs: absent.

**Table 7:** Frequencies^a^ (in cm^−1^) and IR intensities (in km/mol) of various vibrational modes^b^ obtained from the theoretical vibrational spectra of the dipeptides in gas and aqueous phases. Intensities are given in brackets.

Dipeptides	Phases	*ν* (C_4_=O_11_)	*ν* (N_6_–H_10_)	*ν* (C_4_–N_6_)	*ν*_s_ (N_5_–H)	*ν*_as_ (N_5_–H)	Sis (N_5_–H)	*ν* (C_7_–H)	*ν* (C_3_–H)
Sec-Val	Aqueous	1699 (573)	3433 (160)	1552 (673)	3384 (9)	3464 (12)	1667 (59)	2977 (6)	2941 (6)
	Gas	1749 (273)	3469 (57)	1554 (406)	3382 (2)	3463 (7)	1675 (43)	2968 (10)	2937 (2)
Sec-Leu	Aqueous	1696 (533)	3442 (155)	1550 (555)	3383 (8)	3464 (11)	1670 (61)	2975 (9)	2942 (7)
	Gas	1747 (266)	3458 (66)	1559 (359)	3382 (3)	3465 (3)	1676 (44)	2962 (7)	2939 (2)
Sec-Asp	Aqueous	1706 (623)	3415 (169)	1554 (597)	3383 (11)	3464 (13)	1667 (56)	2972 (16)	2941 (6)
	Gas	1758 (260)	3429 (80)	1553 (268)	3376 (9)	3467 (6)	1675 (58)	2951 (24)	2938 (3)
Sec-Ser	Aqueous	1704 (533)	3420 (179)	1550 (555)	3381 (12)	3463 (14)	1668 (58)	3027 (8)	2942 (6)
	Gas	1754 (275)	3454 (76)	1548 (380)	3382 (3)	3464 (3)	1676 (41)	3028 (3)	2940 (2)
Sec-Gln	Aqueous	1697 (628)	3440 (162)	1548 (583)	3381 (9)	3461 (12)	1668 (55)	2970 (21)	2940 (6)
	Gas	1748 (277)	3457 (73)	1551 (447)	3383 (3)	3466 (4)	1676 (42)	2978 (4)	2939 (2)
Sec-His	Aqueous	1704 (507)	3416 (175)	1546 (621)	3384 (12)	3465 (13)	1669 (58)	3027 (8)	2940 (6)
	Gas	1754 (239)	3450 (77)	1548 (413)	3382 (3)	3464 (3)	1676 (43)	3021 (5)	2940 (2)
Sec-Pyl	Aqueous	1698 (537)	3430 (161)	1556 (609)	3384 (10)	3464 (12)	1667 (62)	2976 (4)	2940 (6)
	Gas	1747 (264)	3458 (73)	1560 (380)	3382 (3)	3465 (3)	1676 (41)	2988 (17)	2938 (2)

^a^The frequencies below 1800 cm^−1^ are scaled with 1.01 and for those above 1800 cm^−1^ a correction factor 0.9679 is used.

^b^Vibrational modes: *ν*: stretching; Sis: scissoring; s: symmetric; as: asymmetric.

**Table 8:** HOMO and LUMO energies (eV) and their energy gaps for the dipeptides at B3LYP/6-311++G(d,p) level in gas and aqueous phase.

Dipeptides	Phases	HOMO	LUMO	Energy gap
Sec-Val	Aqueous	−6.3680	−0.5935	5.7745
	Gas	−6.0967	−0.8778	5.2189
Sec-Leu	Aqueous	−6.3699	−0.6171	5.7527
	Gas	−6.0828	−0.8694	5.2134
Sec-Asp	Aqueous	−6.3840	−0.8936	5.4904
	Gas	−6.1067	−0.9009	5.2058
Sec-Ser	Aqueous	−6.3832	−0.8111	5.5721
	Gas	−6.1454	−0.9532	5.1922
Sec-Gln	Aqueous	−6.3786	−0.6713	5.7073
	Gas	−6.0784	−0.8266	5.2518
Sec-His	Aqueous	−6.3826	−0.9107	5.4719
	Gas	−6.1884	−1.0266	5.1617
Sec-Pyl	Aqueous	−6.3696	−0.6525	5.7171
	Gas	−6.0512	−0.7981	5.2532
