# Supplementary material for: Nearest-Neighbor Interactions and Their Influence on the Structural Aspects of Dipeptides
Source: Biochem Res Int. 2013 Sep 18;2013:939865. doi: 10.1155/2013/939865 (PMC3789318; doi:10.1155/2013/939865)
Supplement: Supplementary file 1 — Cartesian coordinates of optimized structures of the dipeptides at b3lyp/6-311++g(d,p) in gas and aqueous phase. [file 939865.f1.pdf]

Supporting information

Nearest-neighbor interactions and their influence on the structural aspects of dipeptides

Gunajyoti Das and Shilpi Mandal

Department of Chemistry, North-Eastern Hill University,

Shillong-793022, Meghalaya, India

* *Author to whom correspondence should be addressed* guna_das@yahoo.co.in Phone: +91-9863334018

Contents:

1. Cartesian coordinates of optimized structures of the dipeptides at b3lyp/6-311++g(d,p) in gas and aqueous phase.

Sec-Val (gas phase)

Center Number	Atomic Number	Atomic Type	Coordinates (Angstroms) X	Y	Z
1	6	0	3.258975	0.686124	-0.768605
2	1	0	4.006059	0.088829	-1.299124
3	6	0	2.117627	-0.307921	-0.420298
4	1	0	1.676058	-0.661684	-1.356708
5	6	0	2.692290	-1.562955	0.242801
6	8	0	3.595984	-2.208393	-0.225428
7	7	0	1.033995	0.295123	0.326645
8	7	0	-0.678010	1.804510	1.767778
9	1	0	-0.650945	2.669050	1.234124
10	6	0	-1.278805	0.712594	0.987274
11	1	0	-1.457260	-0.112954	1.685671
12	6	0	-2.608041	1.070138	0.310104
13	6	0	-0.267570	0.165894	-0.039254
14	1	0	-3.266062	1.548609	1.033509
15	1	0	-2.446819	1.749627	-0.527567
16	8	0	-0.637264	-0.361512	-1.079231
17	1	0	-4.825585	-0.222326	0.370010
18	1	0	-1.209872	1.983622	2.612467
19	1	0	1.199597	0.736109	1.221737
20	8	0	2.089309	-1.889600	1.408456
21	1	0	2.511044	-2.704076	1.723304
22	34	0	-3.592876	-0.516967	-0.389389
23	6	0	2.754509	1.784662	-1.710784
24	6	0	3.923186	1.277939	0.482692
25	1	0	2.321215	1.362828	-2.621543
26	1	0	1.989227	2.399642	-1.228735
27	1	0	3.577213	2.443316	-2.001962
28	1	0	4.767401	1.911176	0.198337
29	1	0	3.228919	1.904694	1.052400
30	1	0	4.308437	0.500184	1.148379

Sec-Val (aqueous phase)

Center Number	Atomic Number	Atomic Type	Coordinates (Angstroms) X	Y	Z
1	6	0	3.258707	0.702861	-0.783507
2	1	0	4.015650	0.114970	-1.310544
3	6	0	2.127311	-0.307810	-0.442295
4	1	0	1.716226	-0.683307	-1.382706
5	6	0	2.710733	-1.543933	0.249295
6	8	0	3.551928	-2.247218	-0.263699
7	7	0	1.029946	0.299710	0.280504
8	7	0	-0.643209	1.872186	1.644221
9	1	0	-0.635904	2.695528	1.047615
10	6	0	-1.267920	0.730372	0.965863
11	1	0	-1.493359	-0.014440	1.737108

12	6	0	-2.572663	1.091423	0.241832
13	6	0	-0.268068	0.040830	0.019269
14	1	0	-3.219582	1.634407	0.926814
15	1	0	-2.374697	1.714642	-0.630419
16	8	0	-0.652699	-0.709954	-0.879036
17	1	0	-4.909304	0.010649	0.167175
18	1	0	-1.160156	2.114310	2.482705
19	1	0	1.198287	0.922550	1.062117
20	8	0	2.208845	-1.777141	1.472325
21	1	0	2.633777	-2.575488	1.826489
22	34	0	-3.627925	-0.493318	-0.362789
23	6	0	2.745811	1.789458	-1.735878
24	6	0	3.912347	1.309672	0.466603
25	1	0	2.324394	1.354443	-2.646382
26	1	0	1.973236	2.401532	-1.262436
27	1	0	3.565576	2.451586	-2.026202
28	1	0	4.753037	1.943673	0.174814
29	1	0	3.215233	1.939731	1.028353
30	1	0	4.297748	0.542011	1.143445

Sec-Leu (gas phase)

Center Number	Atomic Number	Atomic Type	Coordinates (Angstroms) X	Y	Z
1	6	0	3.228218	0.044077	0.096918
2	1	0	4.034786	-0.650984	-0.146225
3	6	0	1.892650	-0.693969	-0.150642
4	1	0	1.609180	-0.590860	-1.202613
5	6	0	2.088590	-2.204921	0.040978
6	8	0	2.908968	-2.848768	-0.562816
7	7	0	0.799628	-0.155287	0.631821
8	7	0	-0.882725	1.128225	2.291698
9	1	0	-0.693875	2.089350	2.020474
10	6	0	-1.515723	0.378262	1.196259
11	1	0	-1.891253	-0.556784	1.626952
12	6	0	-2.687246	1.109164	0.527420
13	6	0	-0.464696	-0.048647	0.153571
14	1	0	-3.366825	1.476981	1.294215
15	1	0	-2.329667	1.952912	-0.064337
16	8	0	-0.776405	-0.282973	-1.006669
17	1	0	-5.054093	0.289782	-0.027659
18	1	0	-1.482520	1.153946	3.108883
19	1	0	0.923170	0.052420	1.614729
20	8	0	1.253886	-2.752114	0.951257
21	1	0	1.444082	-3.702700	0.970100
22	34	0	-3.771061	-0.056606	-0.675445
23	6	0	3.447585	1.330896	-0.723192
24	1	0	3.368948	1.052313	-1.782339
25	1	0	3.316872	0.266427	1.168883
26	6	0	2.414647	2.432471	-0.449390
27	6	0	4.871119	1.854339	-0.482128

28	1	0	5.074957	2.733130	-1.100194
29	1	0	5.009079	2.146813	0.564800
30	1	0	5.622933	1.096101	-0.719146
31	1	0	1.402221	2.119842	-0.710174
32	1	0	2.420272	2.720551	0.608033
33	1	0	2.647034	3.325597	-1.036823

Sec-Leu (aqueous phase)

Center Number	Atomic Number	Atomic Type	Coordinates (Angstroms) X	Y	Z
1	6	0	3.256575	-0.107146	0.103042
2	1	0	3.984978	-0.903152	-0.072697
3	6	0	1.853324	-0.696352	-0.175676
4	1	0	1.661368	-0.674020	-1.251138
5	6	0	1.851529	-2.184674	0.188003
6	8	0	2.300386	-3.042767	-0.537845
7	7	0	0.805782	0.049517	0.495343
8	7	0	-0.766175	1.787229	1.784576
9	1	0	-0.603111	2.612038	1.212740
10	6	0	-1.472108	0.747501	1.026576
11	1	0	-1.859777	0.026793	1.754974
12	6	0	-2.650457	1.290093	0.204728
13	6	0	-0.492484	-0.051119	0.149721
14	1	0	-3.278842	1.901417	0.848265
15	1	0	-2.296218	1.897882	-0.628124
16	8	0	-0.889852	-0.753653	-0.782525
17	1	0	-5.094744	0.544135	-0.124317
18	1	0	-1.317658	2.081389	2.583482
19	1	0	1.012129	0.632691	1.297672
20	8	0	1.345071	-2.442392	1.404245
21	1	0	1.408799	-3.398025	1.564272
22	34	0	-3.847009	-0.129331	-0.535239
23	6	0	3.662610	1.099323	-0.768435
24	1	0	3.558551	0.789233	-1.816222
25	1	0	3.331035	0.150588	1.166676
26	6	0	2.793481	2.346366	-0.554999
27	6	0	5.142827	1.427310	-0.521869
28	1	0	5.471150	2.246379	-1.167700
29	1	0	5.307171	1.735339	0.516489
30	1	0	5.783228	0.563210	-0.721254
31	1	0	1.748246	2.170417	-0.815187
32	1	0	2.831058	2.676283	0.489216
33	1	0	3.157088	3.170106	-1.176017

Sec-Asp (gas phase)

Center Number	Atomic Number	Atomic Type	Coordinates (Angstroms) X	Y	Z

1	6	0	3.005351	0.842644	-0.030494
2	6	0	2.174162	-0.442387	-0.255588
3	1	0	1.775318	-0.450305	-1.276103
4	6	0	3.071284	-1.676739	-0.130647
5	8	0	4.270871	-1.647809	-0.018320
6	7	0	1.023598	-0.520494	0.617134
7	7	0	-0.666219	0.430375	2.472385
8	1	0	-0.425742	1.408642	2.330499
9	6	0	-1.292383	-0.145217	1.274067
10	1	0	-1.589533	-1.169774	1.524731
11	6	0	-2.527606	0.612327	0.771282
12	6	0	-0.234864	-0.259281	0.160658
13	1	0	-3.217494	0.771447	1.598246
14	1	0	-2.246024	1.578890	0.353668
15	8	0	-0.512401	-0.132356	-1.021594
16	1	0	-4.799500	-0.409433	0.139196
17	1	0	-1.279871	0.365069	3.276638
18	1	0	1.142566	-0.428163	1.620162
19	8	0	2.361263	-2.819478	-0.193178
20	1	0	2.991307	-3.554194	-0.139582
21	34	0	-3.542564	-0.373588	-0.634675
22	1	0	3.376407	0.864287	0.995598
23	1	0	3.858287	0.840988	-0.707787
24	6	0	2.156733	2.072880	-0.252673
25	8	0	1.439082	2.583043	0.575031
26	8	0	2.265153	2.541888	-1.514213
27	1	0	1.652000	3.287709	-1.604389

Sec-Asp (aqueous phase)

Center Number	Atomic Number	Atomic Type	Coordinates (Angstroms) X	Y	Z
1	6	0	3.268161	0.419955	0.014196
2	6	0	2.087682	-0.540585	-0.205345
3	1	0	1.748672	-0.467112	-1.242782
4	6	0	2.560248	-1.993280	-0.049301
5	8	0	3.624770	-2.390721	-0.462365
6	7	0	0.957168	-0.195451	0.627674
7	7	0	-0.759363	0.781547	2.421868
8	1	0	-0.639839	1.778627	2.262505
9	6	0	-1.372943	0.126984	1.260858
10	1	0	-1.719323	-0.856934	1.596168
11	6	0	-2.570880	0.895170	0.685484
12	6	0	-0.318406	-0.166597	0.178743
13	1	0	-3.253829	1.145170	1.493996
14	1	0	-2.246534	1.813698	0.196438
15	8	0	-0.637480	-0.384924	-0.989044
16	1	0	-4.944546	0.177905	-0.017301
17	1	0	-1.347266	0.676293	3.241707
18	1	0	1.077200	-0.012946	1.618866

19	8	0	1.657290	-2.780534	0.549042
20	1	0	2.008243	-3.685882	0.571011
21	34	0	-3.644596	-0.155472	-0.631592
22	1	0	3.589299	0.413996	1.056567
23	1	0	4.110794	0.081822	-0.594390
24	6	0	2.969753	1.851513	-0.368953
25	8	0	3.259031	2.817149	0.299920
26	8	0	2.369303	1.948123	-1.570123
27	1	0	2.223753	2.887026	-1.768972

Sec-Ser (gas phase)

Center Number	Atomic Number	Atomic Type	Coordinates (Angstroms) X	Y	Z
1	6	0	2.889395	-0.408555	-1.459645
2	1	0	3.198114	0.612811	-1.714069
3	6	0	2.226573	-0.411730	-0.075354
4	1	0	1.851824	-1.410394	0.143652
5	6	0	3.219220	-0.049952	1.026998
6	8	0	3.546162	-0.757019	1.939198
7	7	0	1.101689	0.509878	-0.037100
8	7	0	-0.674401	2.498653	-0.475175
9	1	0	-0.689703	2.550728	-1.490128
10	6	0	-1.231600	1.224346	0.003154
11	1	0	-1.391834	1.333620	1.081540
12	6	0	-2.562386	0.828258	-0.648249
13	6	0	-0.189845	0.096712	-0.147975
14	1	0	-3.246456	1.674614	-0.617269
15	1	0	-2.411936	0.531212	-1.686860
16	8	0	-0.522312	-1.066028	-0.318637
17	1	0	-4.729879	0.078807	0.499404
18	1	0	-1.207990	3.285256	-0.121897
19	1	0	1.258518	1.509414	0.009854
20	8	0	3.687111	1.216992	0.864992
21	1	0	4.300220	1.394789	1.594046
22	34	0	-3.481339	-0.679748	0.277933
23	1	0	2.144965	-0.749243	-2.188633
24	8	0	4.006742	-1.286178	-1.399751
25	1	0	4.448909	-1.296515	-2.253429

Sec-Ser (aqueous phase)

Center Number	Atomic Number	Atomic Type	Coordinates (Angstroms) X	Y	Z
1	6	0	3.031419	0.133591	-1.492741
2	1	0	3.354127	1.171866	-1.362087
3	6	0	2.247207	-0.329291	-0.257792
4	1	0	1.872708	-1.338541	-0.420610

5	6	0	3.142489	-0.382748	0.981445
6	8	0	3.436413	-1.390791	1.577448
7	7	0	1.104011	0.533973	-0.024159
8	7	0	-0.665237	2.527341	0.076913
9	1	0	-0.703866	2.797700	-0.902695
10	6	0	-1.222297	1.184647	0.279768
11	1	0	-1.409925	1.074817	1.353468
12	6	0	-2.539698	0.950850	-0.472013
13	6	0	-0.178458	0.103887	-0.059834
14	1	0	-3.219754	1.772196	-0.258661
15	1	0	-2.370746	0.899091	-1.547650
16	8	0	-0.510423	-1.054386	-0.305733
17	1	0	-4.809898	-0.046352	0.228448
18	1	0	-1.195251	3.216170	0.599800
19	1	0	1.231238	1.523035	0.165013
20	8	0	3.566045	0.842632	1.339653
21	1	0	4.117541	0.766341	2.134951
22	34	0	-3.501568	-0.711594	0.078894
23	1	0	2.366741	0.077258	-2.361004
24	8	0	4.152714	-0.735504	-1.636368
25	1	0	4.707004	-0.409825	-2.353640

Sec-Gln (gas phase)

Center Number	Atomic Number	Atomic Type	Coordinates (Angstroms) X	Y	Z
1	6	0	-2.660443	-0.444646	0.144904
2	1	0	-2.909601	-0.544126	1.206916
3	6	0	-1.358627	0.370763	-0.009863
4	1	0	-1.166630	0.563743	-1.068019
5	6	0	-1.508779	1.756284	0.626096
6	8	0	-2.141474	2.654898	0.128992
7	7	0	-0.239137	-0.386170	0.510569
8	7	0	1.511504	-2.188721	1.451547
9	1	0	1.358950	-2.930489	0.773719
10	6	0	2.105880	-1.000030	0.823697
11	1	0	2.450193	-0.349386	1.635407
12	6	0	3.298711	-1.301310	-0.094480
13	6	0	1.031318	-0.186292	0.078907
14	1	0	3.994864	-1.957091	0.425523
15	1	0	2.967148	-1.791612	-1.010893
16	8	0	1.324855	0.591362	-0.818864
17	1	0	5.634224	-0.250126	-0.203233
18	1	0	2.113800	-2.557552	2.178994
19	1	0	-0.359080	-1.028573	1.283753
20	8	0	-0.901921	1.865085	1.827202
21	1	0	-1.061504	2.767271	2.144454
22	34	0	4.335090	0.322080	-0.614739
23	1	0	-2.462662	-1.454060	-0.220638
24	6	0	-5.074319	-0.783069	-0.532546
25	8	0	-5.001309	-1.966602	-0.250079

26	7	0	-6.264273	-0.175568	-0.817522
27	1	0	-7.098870	-0.740430	-0.845843
28	1	0	-6.324942	0.796695	-1.069792
29	6	0	-3.853863	0.130218	-0.618413
30	1	0	-3.600255	0.234852	-1.680511
31	1	0	-4.105834	1.133897	-0.268685

Sec-Gln (aqueous phase)

Center Number	Atomic Number	Atomic Type	Coordinates (Angstroms) X	Y	Z
1	6	0	-2.697873	-0.460932	0.095324
2	1	0	-2.827400	-0.568846	1.176703
3	6	0	-1.372095	0.273551	-0.188243
4	1	0	-1.267384	0.431839	-1.264340
5	6	0	-1.380232	1.675743	0.426757
6	8	0	-1.744121	2.664188	-0.168295
7	7	0	-0.265112	-0.537641	0.284799
8	7	0	1.451609	-2.491693	0.928357
9	1	0	1.374290	-3.065765	0.092488
10	6	0	2.060178	-1.191120	0.625008
11	1	0	2.338862	-0.738042	1.582920
12	6	0	3.317653	-1.289816	-0.249936
13	6	0	1.020204	-0.238205	0.011723
14	1	0	3.990655	-2.029769	0.176907
15	1	0	3.063125	-1.585150	-1.267800
16	8	0	1.351776	0.746960	-0.650390
17	1	0	5.659910	-0.229479	-0.043249
18	1	0	2.020669	-3.004534	1.593388
19	1	0	-0.427634	-1.373288	0.834499
20	8	0	-0.997410	1.689597	1.713718
21	1	0	-1.064410	2.600321	2.043403
22	34	0	4.365121	0.408539	-0.350167
23	1	0	-2.608090	-1.466242	-0.321193
24	6	0	-5.177851	-0.642020	-0.385595
25	8	0	-5.140171	-1.870685	-0.433798
26	7	0	-6.337406	0.039587	-0.242072
27	1	0	-7.210227	-0.466636	-0.214091
28	1	0	-6.367685	1.046045	-0.208198
29	6	0	-3.924672	0.217643	-0.517228
30	1	0	-3.766104	0.375409	-1.590540
31	1	0	-4.098807	1.202490	-0.079517

Sec-His (gas phase)

Center Number	Atomic Number	Atomic Type	Coordinates (Angstroms) X	Y	Z
1	7	0	2.014412	2.032728	1.610253

2	1	0	1.892414	1.606634	2.525007
3	6	0	2.411701	1.033827	0.605702
4	1	0	2.748514	1.588573	-0.277026
5	6	0	3.541581	0.098520	1.052439
6	6	0	1.181324	0.227149	0.141843
7	1	0	4.356220	0.688865	1.468336
8	1	0	3.188334	-0.602292	1.809875
9	8	0	1.277507	-0.926934	-0.245470
10	1	0	5.697406	-0.493272	-0.206240
11	1	0	2.719623	2.755445	1.703796
12	34	0	4.319820	-0.974520	-0.437445
13	7	0	0.013251	0.924992	0.171360
14	1	0	0.041783	1.838794	0.607795
15	6	0	-1.251245	0.365667	-0.284982
16	1	0	-1.011586	-0.512075	-0.883804
17	6	0	-2.147841	-0.030126	0.905912
18	6	0	-1.913347	1.376931	-1.220338
19	1	0	-1.569956	-0.739977	1.509289
20	1	0	-2.311778	0.858473	1.525654
21	6	0	-3.464368	-0.625643	0.510620
22	8	0	-2.254093	2.511584	-0.554729
23	8	0	-2.093547	1.229094	-2.398092
24	7	0	-4.518096	-0.720372	1.403708
25	6	0	-3.942170	-1.196483	-0.644716
26	1	0	-4.518569	-0.396220	2.358245
27	6	0	-5.555962	-1.332861	0.755780
28	7	0	-5.240761	-1.630085	-0.480060
29	1	0	-3.432632	-1.312763	-1.588180
30	1	0	-6.503233	-1.527599	1.235593
31	1	0	-2.649017	3.118420	-1.199261

Sec-His (aqueous phase)

Center Number	Atomic Number	Atomic Type	Coordinates (Angstroms) X	Y	Z
1	7	0	1.939827	2.018046	1.628161
2	1	0	1.867632	1.577957	2.542024
3	6	0	2.381784	1.057609	0.610359
4	1	0	2.690476	1.639937	-0.264892
5	6	0	3.563663	0.187503	1.057994
6	6	0	1.199482	0.196353	0.126315
7	1	0	4.345421	0.828116	1.459132
8	1	0	3.256856	-0.522805	1.825672
9	8	0	1.375191	-0.900448	-0.400870
10	1	0	5.785903	-0.417517	-0.101212
11	1	0	2.605038	2.779191	1.713044
12	34	0	4.411861	-0.845997	-0.426320
13	7	0	-0.014965	0.768876	0.298920
14	1	0	-0.015373	1.657149	0.790153
15	6	0	-1.266214	0.153690	-0.114984
16	1	0	-1.031764	-0.850236	-0.464614

17	6	0	-2.258674	0.085734	1.070761
18	6	0	-1.813142	0.913462	-1.326975
19	1	0	-1.790255	-0.531894	1.839625
20	1	0	-2.380567	1.086717	1.494126
21	6	0	-3.593194	-0.496211	0.727839
22	8	0	-2.297650	2.123706	-0.985849
23	8	0	-1.798318	0.500904	-2.461549
24	7	0	-4.643773	0.248600	0.224166
25	6	0	-4.083307	-1.778412	0.799541
26	1	0	-4.636555	1.243825	0.050470
27	6	0	-5.693810	-0.592245	0.021503
28	7	0	-5.389921	-1.829900	0.358867
29	1	0	-3.566564	-2.659646	1.148683
30	1	0	-6.641484	-0.250674	-0.365422
31	1	0	-2.583548	2.586226	-1.790675

Sec-Pyl (gas phase)

Center Number	Atomic Number	Atomic Type	Coordinates (Angstroms) X	Y	Z
1	6	0	-7.579984	-0.266790	-1.400796
2	6	0	-5.968700	-0.066563	0.136175
3	6	0	-6.793340	-1.308557	0.583499
4	6	0	-8.049256	-1.199930	-0.313632
5	1	0	-6.140291	0.771405	0.826396
6	1	0	-6.232711	-2.202102	0.290635
7	1	0	-8.398468	-2.159496	-0.705053
8	1	0	-8.897121	-0.748487	0.219725
9	7	0	-6.484824	0.343417	-1.185424
10	1	0	-8.136737	-0.089662	-2.319873
11	6	0	-7.069479	-1.378906	2.083943
12	1	0	-6.131420	-1.424184	2.639873
13	1	0	-7.627637	-0.498572	2.421210
14	1	0	-7.659809	-2.265279	2.334598
15	6	0	-4.454475	-0.296124	0.141132
16	8	0	-3.904783	-0.835510	1.094714
17	7	0	-3.802058	0.149029	-0.959151
18	1	0	-4.369345	0.587870	-1.672377
19	6	0	-2.354860	0.068917	-1.103348
20	1	0	-2.021868	-0.826784	-0.574529
21	1	0	-2.126732	-0.065861	-2.164821
22	6	0	-1.622066	1.299092	-0.551864
23	1	0	-1.968964	2.194427	-1.081426
24	1	0	-1.900727	1.424093	0.499738
25	6	0	-0.100415	1.173257	-0.677035
26	1	0	0.177103	1.032315	-1.728544
27	1	0	0.232280	0.273273	-0.148851
28	6	0	0.638137	2.395100	-0.123912
29	1	0	0.356244	3.288900	-0.687780
30	1	0	0.344357	2.569427	0.918692
31	6	0	2.177805	2.257410	-0.194441

32	1	0	2.465883	2.016259	-1.220133
33	6	0	2.810467	3.610934	0.118813
34	8	0	2.739976	4.567403	-0.609594
35	7	0	2.684060	1.186751	0.641137
36	7	0	3.109261	-0.767335	2.446870
37	1	0	2.240574	-1.288958	2.366622
38	6	0	3.902963	-0.849584	1.212302
39	1	0	4.900143	-0.462843	1.451018
40	6	0	4.056309	-2.268717	0.649759
41	6	0	3.337684	0.106764	0.142600
42	1	0	4.364788	-2.942278	1.447402
43	1	0	3.113943	-2.623502	0.230653
44	8	0	3.504243	-0.103487	-1.051356
45	1	0	6.183707	-3.493132	-0.098420
46	1	0	3.621098	-1.146071	3.235950
47	1	0	2.604473	1.237186	1.649253
48	8	0	3.423643	3.652576	1.325601
49	1	0	3.763573	4.553311	1.440256
50	34	0	5.441448	-2.411764	-0.778480

Sec-Pyl (aqueous phase)

Center Number	Atomic Number	Atomic Type	Coordinates (Angstroms) X	Y	Z
1	6	0	-7.868806	0.294671	-1.334473
2	6	0	-6.322377	-0.113853	0.233736
3	6	0	-7.232821	-1.381432	0.225943
4	6	0	-8.424086	-0.921730	-0.647800
5	1	0	-6.476270	0.455031	1.160253
6	1	0	-6.701588	-2.177063	-0.305920
7	1	0	-8.769314	-1.677858	-1.357366
8	1	0	-9.293414	-0.634066	-0.042110
9	7	0	-6.761407	0.740664	-0.889573
10	1	0	-8.376216	0.797460	-2.155719
11	6	0	-7.623271	-1.891343	1.612359
12	1	0	-6.739859	-2.184062	2.183292
13	1	0	-8.153687	-1.116971	2.176158
14	1	0	-8.281592	-2.761367	1.535253
15	6	0	-4.826297	-0.431535	0.189568
16	8	0	-4.326565	-1.206056	1.010939
17	7	0	-4.129930	0.183498	-0.785984
18	1	0	-4.652180	0.821040	-1.373557
19	6	0	-2.690532	0.043609	-0.974339
20	1	0	-2.398562	-0.931395	-0.580348
21	1	0	-2.492207	0.041963	-2.049617
22	6	0	-1.882831	1.154130	-0.290153
23	1	0	-2.211781	2.126317	-0.674493
24	1	0	-2.104656	1.144749	0.782471
25	6	0	-0.375469	0.991141	-0.512098
26	1	0	-0.160016	0.999887	-1.587333
27	1	0	-0.057468	0.012195	-0.138378

28	6	0	0.436770	2.098523	0.168629
29	1	0	0.129375	3.071815	-0.222420
30	1	0	0.232970	2.110025	1.245774
31	6	0	1.957303	1.960487	-0.054137
32	1	0	2.156168	1.882286	-1.125440
33	6	0	2.656612	3.249598	0.386527
34	8	0	2.601008	4.277826	-0.249491
35	7	0	2.475795	0.767966	0.584622
36	7	0	2.894489	-1.540200	1.860483
37	1	0	2.249958	-2.132514	1.343113
38	6	0	4.000092	-1.088245	1.007585
39	1	0	4.783218	-0.707669	1.672564
40	6	0	4.596477	-2.208657	0.143283
41	6	0	3.574038	0.117419	0.151117
42	1	0	4.817400	-3.064586	0.776720
43	1	0	3.896470	-2.513114	-0.635041
44	8	0	4.221801	0.460633	-0.839766
45	1	0	6.997628	-2.984531	-0.373480
46	1	0	3.245853	-2.087603	2.638778
47	1	0	2.050034	0.403717	1.429803
48	8	0	3.308088	3.142231	1.554943
49	1	0	3.698887	4.005819	1.766220
50	34	0	6.322786	-1.723434	-0.738926
